# Antegrade Hybrid Chimney TEVAR Endograft in a Patient with Blunt Aortic Injury: A Challenging Case with Technical Success but Unfavorable Result

**DOI:** 10.1155/2021/6380428

**Published:** 2021-04-10

**Authors:** Fotios Eforakopoulos, Maria Giovani, Petros Zampakis, Christina Kalogeropoulou, Fotini Fligou, Nikolaos Charoulis, Efstratios Koletsis, Dimitrios Dougenis

**Affiliations:** ^1^Department of Cardiothoracic Surgery, University of Patras, Patras, Greece; ^2^Department of Pediatric Surgery, Mitera Children's Hospital, Marousi, Athens, Greece; ^3^Department of Radiology, University of Patras, Patras, Greece; ^4^Department of Anesthesiology and Intensive Care Medicine, University of Patras, Patras, Greece; ^5^Department of Cardiac Surgery, School of Medicine, National and Kapodistrian University of Athens, Attikon University Hospital, Athens, Greece

## Abstract

Thoracic Endovascular Aortic Repair (TEVAR) has modified aortic medicine, particularly in patients with traumatic aortic injury (TAI). Conventional repair of TAI in the aortic arch is technically demanding as it requires cardiopulmonary bypass and deep hypothermic arrest with still a significant number of complications. Despite recent improvements in endovascular techniques, many patients have been excluded from endovascular repair due to unfavorable anatomy. To increase the feasibility of endovascular repair, adjunctive open extra-anatomical bypasses may be required to provide an adequate proximal landing zone. Several methods, for instance, chimney technique, hybrid technique, and fenestrated or branched stent-grafts, have been proposed as options to preserve the supra-aortic branches, each with its own advantages and disadvantages. We herein present a patient with complex anatomical features and blunt aortic injury, who underwent antegrade chimney stent-graft deployment through the ascending aorta, not otherwise amenable to standard retrograde delivery because of severe peripheral artery disease. The remarkable aspect, in this case, is that both stents were placed antegrade, through the ascending aorta.

## 1. Introduction

Traumatic aortic injury occurs in 2% of cases with blunt thoracic trauma and is considered a life-threatening condition [1]. The main cause of TAI is the movement of the mobile aortic arch against the fixed descending thoracic aorta during deceleration injury. There is a predilection for endovascular repair when anatomically possible and when institutional resources are available to support the attempt [2]. Clinical and anatomic features that support the use of TEVAR require multiple severe injuries or comorbidities with a limited life expectancy and lesions confined to the distal arch or the descending thoracic aorta. Similarly, because of severe chest or lung injuries, the patient may not tolerate one-lung ventilation, which is demanded for open surgery.

Occasionally, when supra-aortic vessels are involved, fenestrated custom-made grafts are used. These stent-grafts had to be based on the patients' individual anatomy and were thus time-consuming, limiting their application in an urgent setting. Combined hybrid techniques (both endovascular and surgical) became popular providing the possibility for arch rerouting, without cardiopulmonary bypass and hypothermic arrest. We herein present a case of a patient with a traumatic aortic transection located across the origin of the LSA who required immediate LSA revascularization because of ischemia on the left arm. The unique feature of our work is that both grafts in the descending aorta and in the LSA were placed antegrade via the ascending aorta after partial sternotomy, because of multiple atherosclerotic lesions in femoral and iliac vessels.

## 2. Case Report

**Figure 1:**
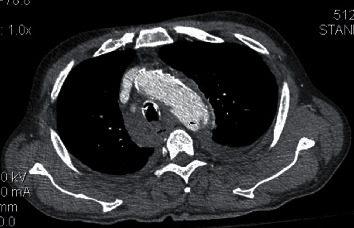
Preoperative CTA showed transection of the distal aortic arch.

A 79-year-old male with COPD and peripheral artery disease was transferred to our institution from a secondary hospital with the diagnosis of aortic transection, after a lone motorcyclist accident without wearing a helmet. While arriving at our emergency room, the patient was confused and complained of chest pain especially aggravated with deep breathing. Physical examinations showed stable vital signs: blood pressure (BP), 122/74 mmHg; pulse rate, 98/min; and respiratory rate, 20/min. Glasgow Coma Scale at admission was 8, so he was intubated on arrival. His injuries included subarachnoid and subdural hemorrhage, fracture of the occipital bone, mandibular fracture, rib fractures with bilateral pulmonary contusions, hemothorax in the left side, and fracture of the left femur as well. Blood loss through the chest tube at insertion was 400 ml and stabilized at 700 ml thereafter. Three-dimensional reconstruction images of contrast-enhanced computed tomographic angiography (CTA) demonstrated an intimal injury with periaortic hematoma (grade II aortic injury). It was located across the origin of the left subclavian artery (Figure 1). A multidisciplinary trauma team approach was instituted.

As there is no endograft availability in our institution, orthopedists stabilized the femur, and then, we proceeded to treat the aortic injury given its severity. Fortunately, the patient was hemodynamically stable. Anti-impulse therapy was administered during the whole procedure which was held in the operating room with mobile c-arm. The patient was placed in a supine position. Aortography was performed via a catheter placed percutaneously through the brachial artery to confirm the anatomy and define the landing zones for the device. Because of multiple atherosclerotic lesions in both femoral arteries and in the left iliac artery, extraperitoneal exposure of the right iliac artery was performed. An incision was made 2 cm above and parallel to the inguinal ligament, extending from the lateral edge of the rectus sheath to a point 2 cm cephalad to the anterior superior iliac spine. After exposure of the external iliac artery, it was found that the guidewire could not be advanced in the descending thoracic aorta due to heavily calcified vessels. It was decided to proceed with partial sternotomy in order to place the stent-graft to the proper position in an antegrade fashion.

**Figure 2:**
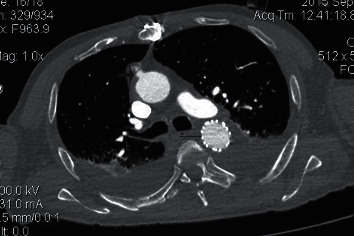
The first arrow indicates a graft anastomosed to the ascending aorta. The second (thin) arrow indicates endograft in the distal arch and the descending thoracic aorta.

**Figure 3:**
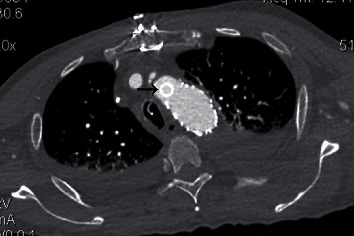
The proximal end of the chimney graft in the aortic arch.

After completion of partial sternotomy, lowering the blood pressure, a partial clamp was placed on the ascending aorta and an 8 mm Goretex thin-walled, ringed graft was anastomosed in end to side fashion to the aorta (Figure 2). An access sheath was placed in the ringed graft, and a guidewire is advanced through the sheath under fluoroscopic guidance from the ascending to the descending aorta. A Conformable Gore TAG stent-graft 34 mm × 10cm was positioned by advancing it over a stiff guidewire (Lunderquist). Its position was confirmed with repeated aortography especially in LAO 30 projection. Before device deployment, the patient's blood pressure was lowered again to lessen the arterial impulse and minimize the potential for misplacement during deployment. The device was then deployed, and completion aortogram was performed to confirm patency of the endograft and successful exclusion of the transection (i.e., no endoleak). However, the LSA was covered and the elimination of arterial pulse with a decrease in oxygen saturation of the left hand was noted. The limb became pale and cold, so a placement of additional Gore Viabahn Endoprosthesis 10 mm × 10cm in the covered LSA was decided (Figure 3). The arterial pulse and oxygen saturation were immediately restored. The guidewires and introducer sheaths were removed, and the arteriotomies were repaired. Transesophageal echocardiography and angiography were used together to position and deploy the device precisely.

**Figure 4:**
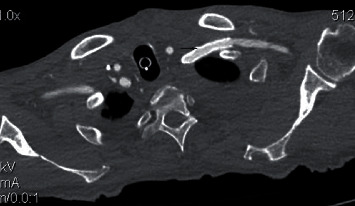
Postoperative CTA showed the proper position of the chimney graft inside the LSA (arrow) and the patency of the vessel.

Subsequently, the patient was admitted to the ICU for deep sedation and ventilator management. Postoperative CTA disclosed appropriate position of stent-grafts and adequate flow to the left arm (Figure 4). On the POD 10, the patient's oxygenation worsened, and purulent sputum with fever made an appearance. We, therefore, arrived at a diagnosis of ventilator-associated pneumonia (VAP), and his PaO_2_/FiO_2_ ratio deteriorated, and multiple organ failure was developed. He required continuous venous-venous hemofiltration for severe acute kidney injury. Finally, the patient deceased on POD 22.

In this patient, we did not administer antiplatelet drugs nor heparin during stent-graft deployment because of the traumatic brain injury.

## 3. Discussion

Patients on whom blunt aortic injury is in close proximity to the origin of the left subclavian artery (<2 cm distance) should undergo intentional coverage of this vessel. This procedure is needed in approximately 20% of cases and is usually well-tolerated [3, 4]. However, total coverage of the LSA increases the incidence of stroke (1,2%), of left upper limb ischemia (4%), and of patients requiring additional procedure (2,9%) [5]. Ischemic lesions and vertebrobasilar insufficiency are usually related to the left vertebral artery diameter < 3 mm [6]. Stroke can present in patients with dominant left vertebral artery and coronary steal syndrome in cases of patients who have a left internal mammary coronary bypass graft. When carotid-subclavian bypass for LSA revascularization is performed prior to its coverage, no instances of stroke, limb ischemia, spinal cord ischemia, endoleak, or death have occurred (hybrid procedure) [7]. Rarely, patients may evolve debilitating arm claudication symptoms later, at which time, an elective extra-anatomic procedure can be performed. Spinal drainage should be performed in cases of extensive coverage of the thoracic aorta, a history of prior open or endovascular aneurysm repair, or presence of internal iliac artery atherosclerotic lesions, with the potential risk for paraplegia [7–12].

Hybrid aortic repair is usually achieved gradually. The first stage is to bypass critical vessels supplying the brain or the left upper limb that will later be covered by the stent-graft, using an open approach. This is in general known as a “debranching” procedure. However, the open repair of the aortic arch remains the “gold standard” but the hybrid repair is an acceptable alternative. When the device is deployed in zone 2 (distal to the left common carotid origin but proximal to the subclavian artery), an extra-anatomical LCCA-LSA bypass or a transposition of the LSA into the LCCA before the positioning of the device would be enough for a safe sealing of the endograft. A substantial alternative to the hybrid approach is the use of fenestrated or chimney grafts.

Chimney graft technique permits the landing zones to be expanded to the appropriate position in the aortic arch in order to safely seal the damaged wall. Compared to the other custom-made devices, these endografts are cheaper and less complex and are proposed in urgent conditions as there is no necessity for previous device customization. Chimney endograft provides adequate blood flow to the LSA preventing both spinal cord ischemia and upper limb ischemia. This procedure can be applied in various occasions such as those with dominant vertebral artery and severe atherosclerotic right vertebral artery, those with congenital right vertebral artery dysplasia, those with left internal mammary artery (LIMA) graft, and those with left arm dialysis access [13]. Frequently, in case that the lesion is located near the LSA at the outer curvature of the arch, endoleak type Ia ensues [14]. Obviously, the radial supporting power of the aortic device is positively related to the patency of the chimney graft. The proximal landing zone of an aortic endograft must be located at the origin of the LSA; otherwise, migration of the main graft with simultaneous occlusion of LCCA or collapse of the chimney graft may be apparent [15]. Consequently, the patency of the chimney graft is affected by the distance between the proximal tip of the aortic stent-graft and the LSA origin. The perfect deployment for both devices is the proximal end of the chimney stent to be exceeded from the proximal aortic stent-graft about 1 cm.

The antegrade stent-graft placement from the ascending aorta that we present here was proved a reliable alternative in the urgent setting with difficulty in accessing the femoral and iliac vessels. An embolic complication was anticipated because of arch manipulation, but fortunately, it did not happen. Given his comorbidities and traumatic injuries, our patient was not a suitable candidate for open surgical repair and was performed endovascular repair applying an antegrade technique. To the best of our knowledge, it is the first time that antegrade implantation of chimney graft in TAI through the ascending aorta has been reported. Several groups have published small series with a transapical approach through the left ventricle to treat acute and chronic pathologies of the ascending aorta [16, 17]. The technical success is promising in selected patients [18]. However, none of them placed a chimney graft antegrade.

Other ways for the antegrade endograft placement that have been reported are the axillary approach through the right standard infraclavicular incision and direct aortic placement with elephant trunk procedure during deep circulatory arrest [19]. There are series with a small number of patients, so a study in a larger population will be required to prove the effectiveness of the antegrade technique. From the existing reports, neurologic complications appeared frequently in the antegrade hybrid approach (0%-25%) [20–23]. It is not the first time that we used antegrade positioning but it was our first attempt for antegrade placement of chimney graft. Unfortunately, the patient died 22 days after surgery because of complications that were not related to the stent-grafts. Our remaining cases will be reported in a future work.

## 4. Conclusion

Hybrid procedure with antegrade stent-graft deployment seems to be a promising treatment for many aortic pathologies including blunt aortic injury. A small number of reported cases and eventual neurologic complications suggest the obligation for further refinement to improve the safety of this technique.
